# Supplementary material for: Repair of noise-induced damage to stereocilia F-actin cores is facilitated by XIRP2 and its novel mechanosensor domain
Source: eLife. 2023 Jun 9;12:e72681. doi: 10.7554/eLife.72681 (PMC10259482; doi:10.7554/eLife.72681)
Supplement: Figure 8—source data 1. [file elife-72681-fig8-data1.zip › Figure 8 - source data/Figure 8 source data legend.pptx]

## Slide 1
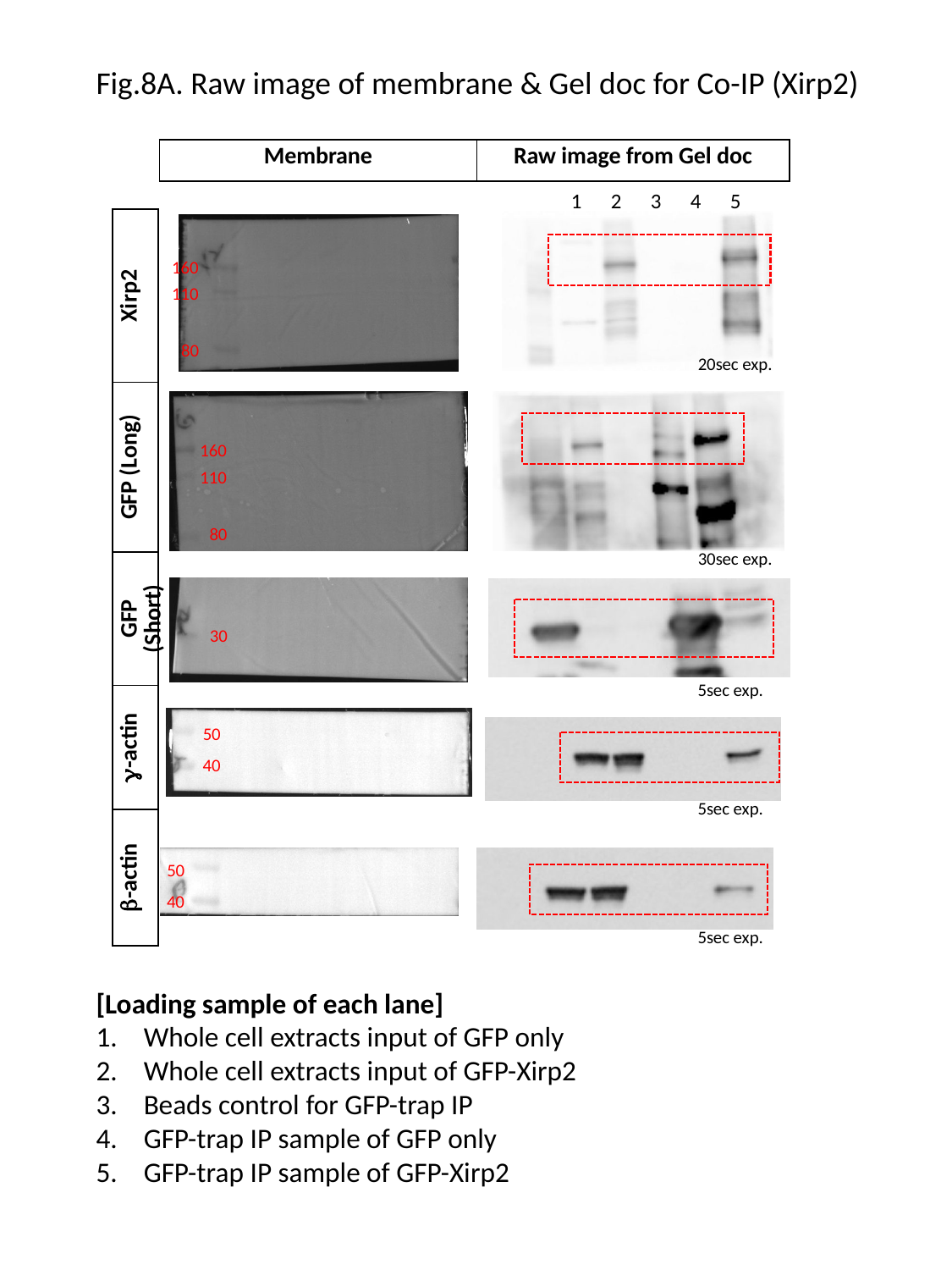

Fig.8A. Raw image of membrane & Gel doc for Co-IP (Xirp2)
| Membrane | Raw image from Gel doc |
| --- | --- |
1 2 3 4 5
| Xirp2 |
| --- |
| GFP (Long) |
| GFP (Short) |
| g-actin |
| b-actin |
160
110
80
20sec exp.
160
110
80
30sec exp.
30
5sec exp.
50
40
5sec exp.
50
40
5sec exp.
[Loading sample of each lane]
Whole cell extracts input of GFP only
Whole cell extracts input of GFP-Xirp2
Beads control for GFP-trap IP
GFP-trap IP sample of GFP only
GFP-trap IP sample of GFP-Xirp2

## Slide 2
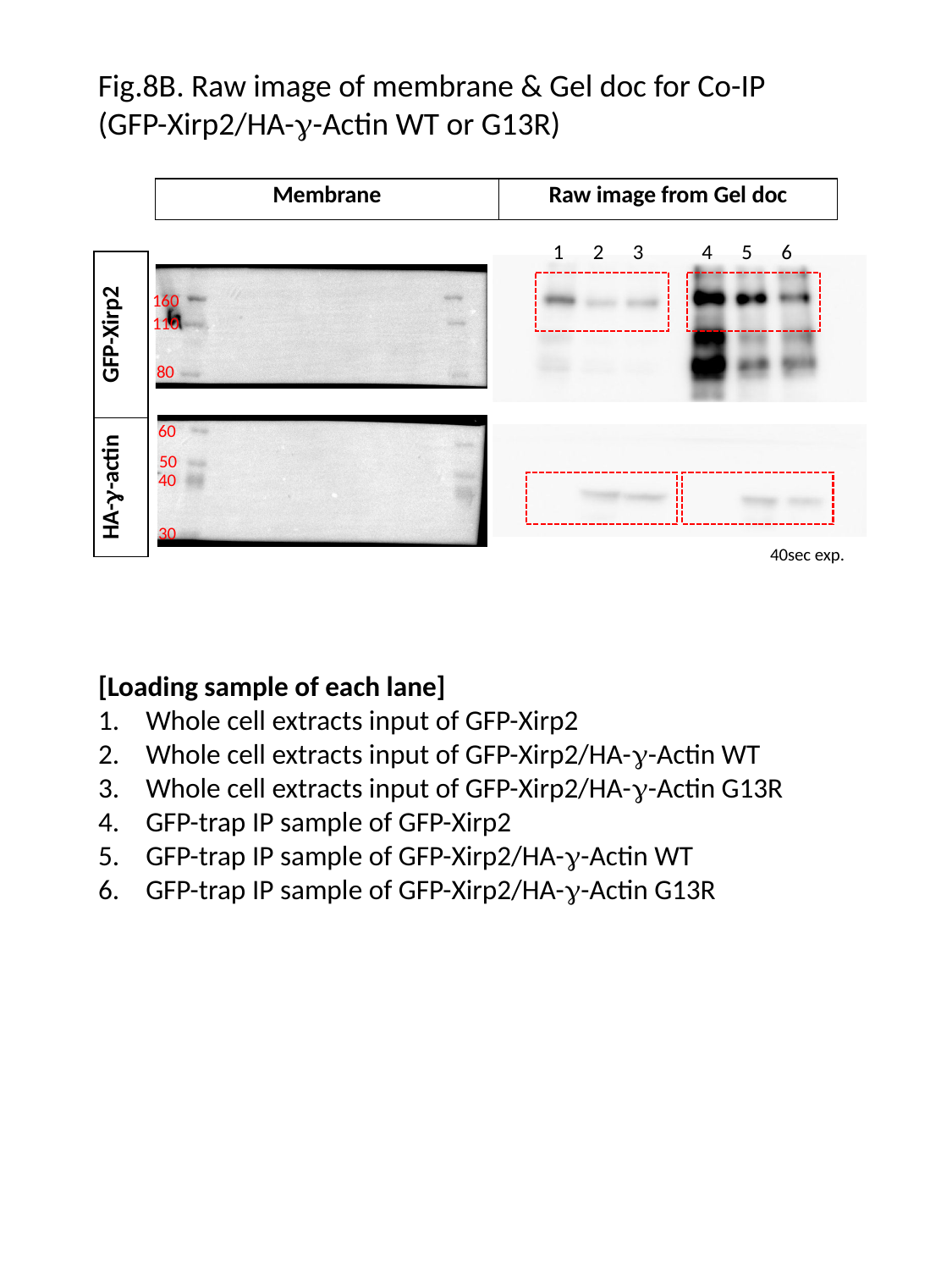

Fig.8B. Raw image of membrane & Gel doc for Co-IP
(GFP-Xirp2/HA-g-Actin WT or G13R)
| Membrane | Raw image from Gel doc |
| --- | --- |
1 2 3 4 5 6
| GFP-Xirp2 |
| --- |
| HA-g-actin |
160
110
80
60
50
40
30
40sec exp.
[Loading sample of each lane]
Whole cell extracts input of GFP-Xirp2
Whole cell extracts input of GFP-Xirp2/HA-g-Actin WT
Whole cell extracts input of GFP-Xirp2/HA-g-Actin G13R
GFP-trap IP sample of GFP-Xirp2
GFP-trap IP sample of GFP-Xirp2/HA-g-Actin WT
GFP-trap IP sample of GFP-Xirp2/HA-g-Actin G13R

## Slide 3
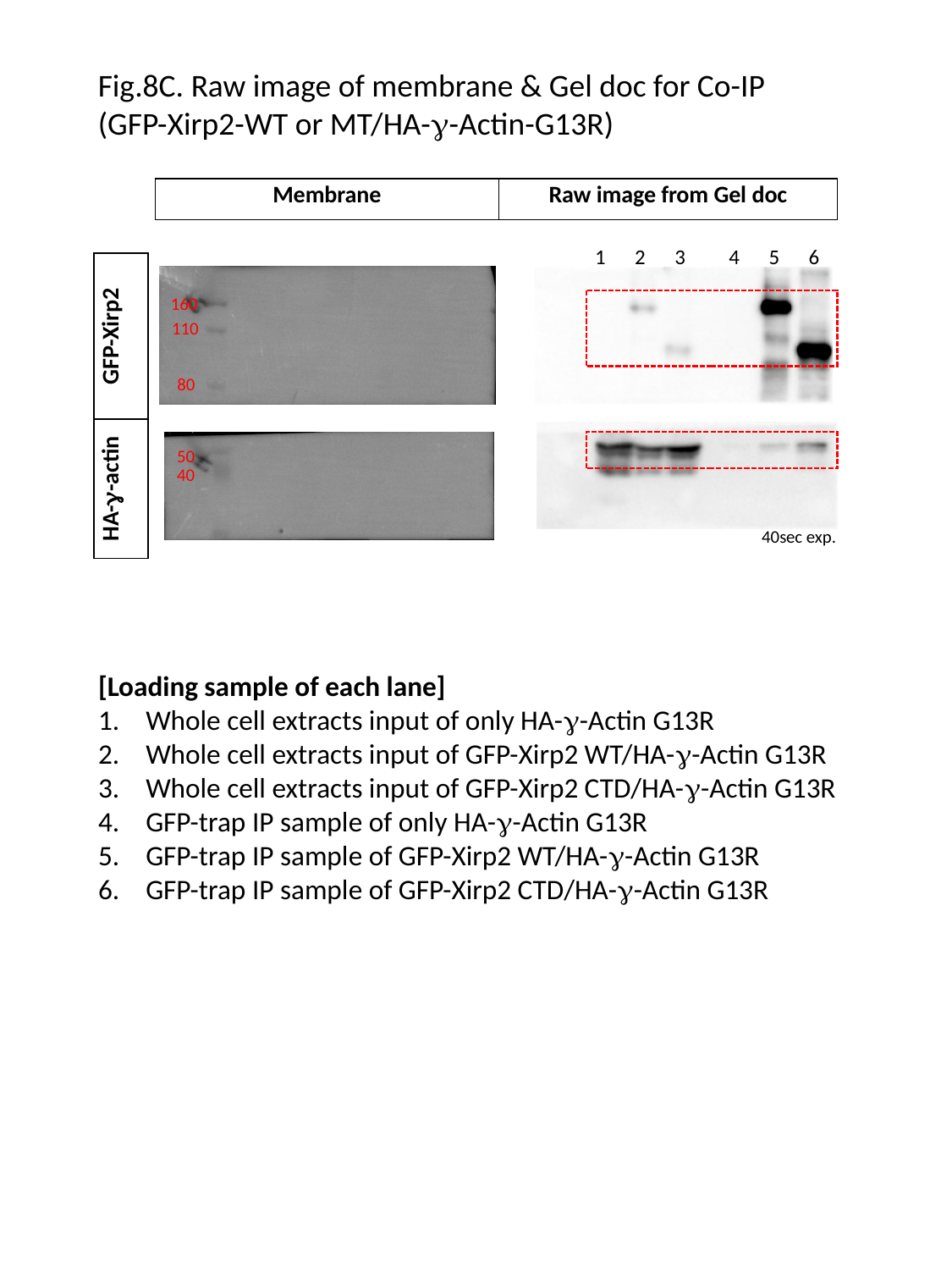

Fig.8C. Raw image of membrane & Gel doc for Co-IP
(GFP-Xirp2-WT or MT/HA-g-Actin-G13R)
| Membrane | Raw image from Gel doc |
| --- | --- |
1 2 3 4 5 6
| GFP-Xirp2 |
| --- |
| HA-g-actin |
160
110
80
50
40
40sec exp.
[Loading sample of each lane]
Whole cell extracts input of only HA-g-Actin G13R
Whole cell extracts input of GFP-Xirp2 WT/HA-g-Actin G13R
Whole cell extracts input of GFP-Xirp2 CTD/HA-g-Actin G13R
GFP-trap IP sample of only HA-g-Actin G13R
GFP-trap IP sample of GFP-Xirp2 WT/HA-g-Actin G13R
GFP-trap IP sample of GFP-Xirp2 CTD/HA-g-Actin G13R
