# Supplementary figures and images for: Repair of noise-induced damage to stereocilia F-actin cores is facilitated by XIRP2 and its novel mechanosensor domain
Source: eLife. 2023 Jun 9;12:e72681. doi: 10.7554/eLife.72681 (PMC10259482; doi:10.7554/eLife.72681)

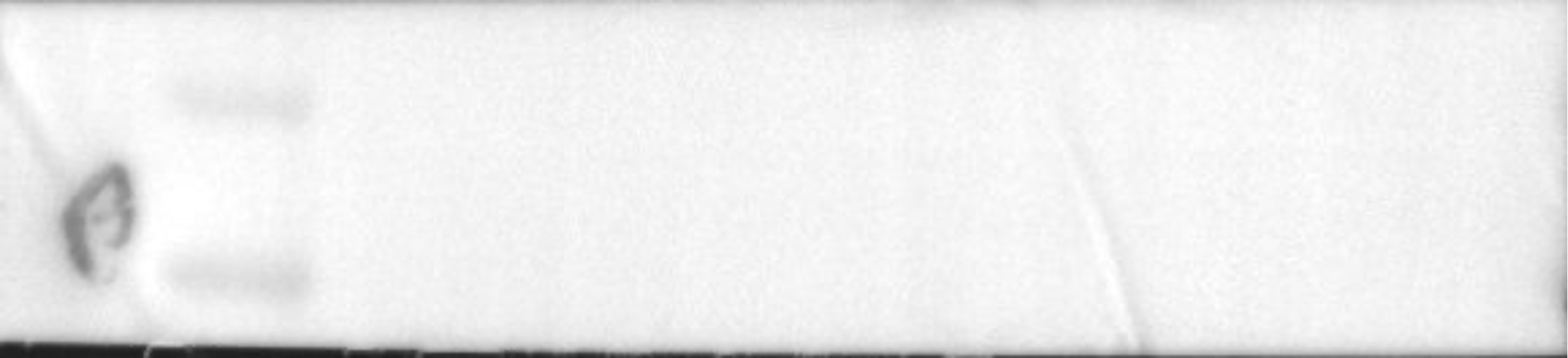

Supplement: Figure 8—source data 1. [file elife-72681-fig8-data1.zip › Figure 8 - source data/Figure 8A - source data 3 bACT membrane.jpg]

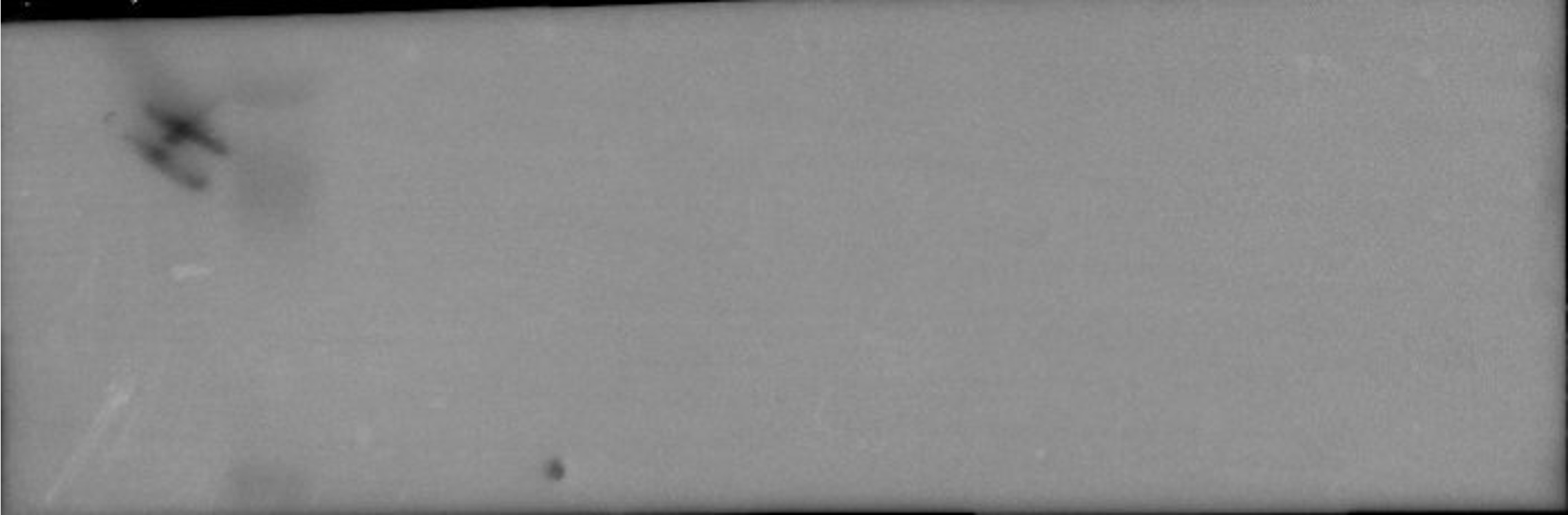

Supplement: Figure 8—source data 1. [file elife-72681-fig8-data1.zip › Figure 8 - source data/Figure 8C - source data 3 HAgACT membrane.jpg]

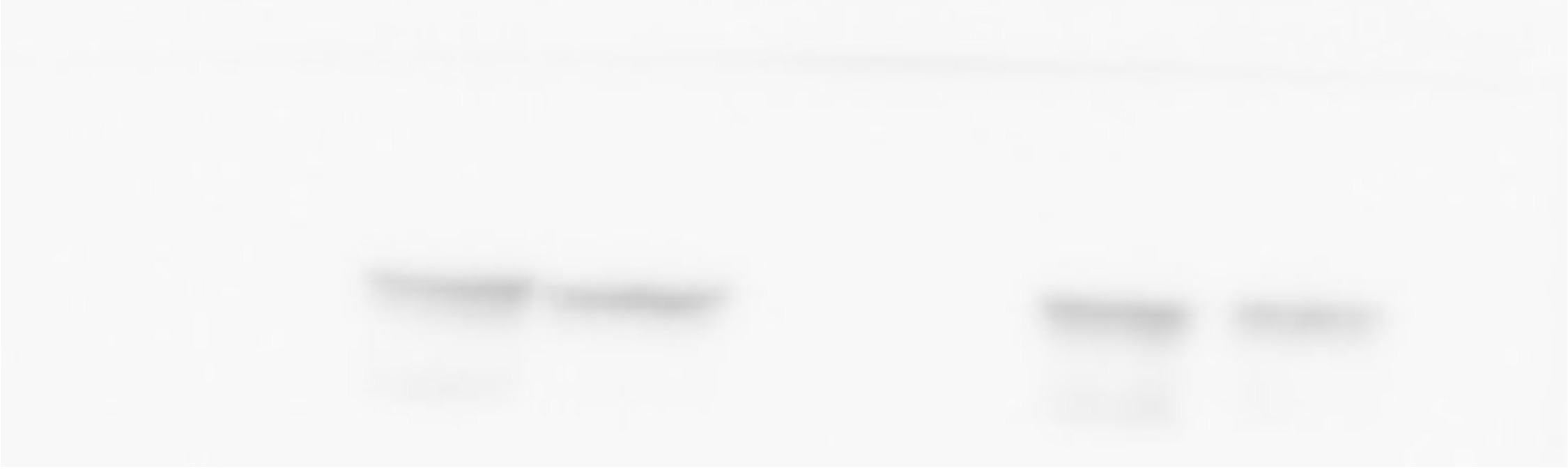

Supplement: Figure 8—source data 1. [file elife-72681-fig8-data1.zip › Figure 8 - source data/Figure 8B - source data 2 HAgACT wb.jpg]

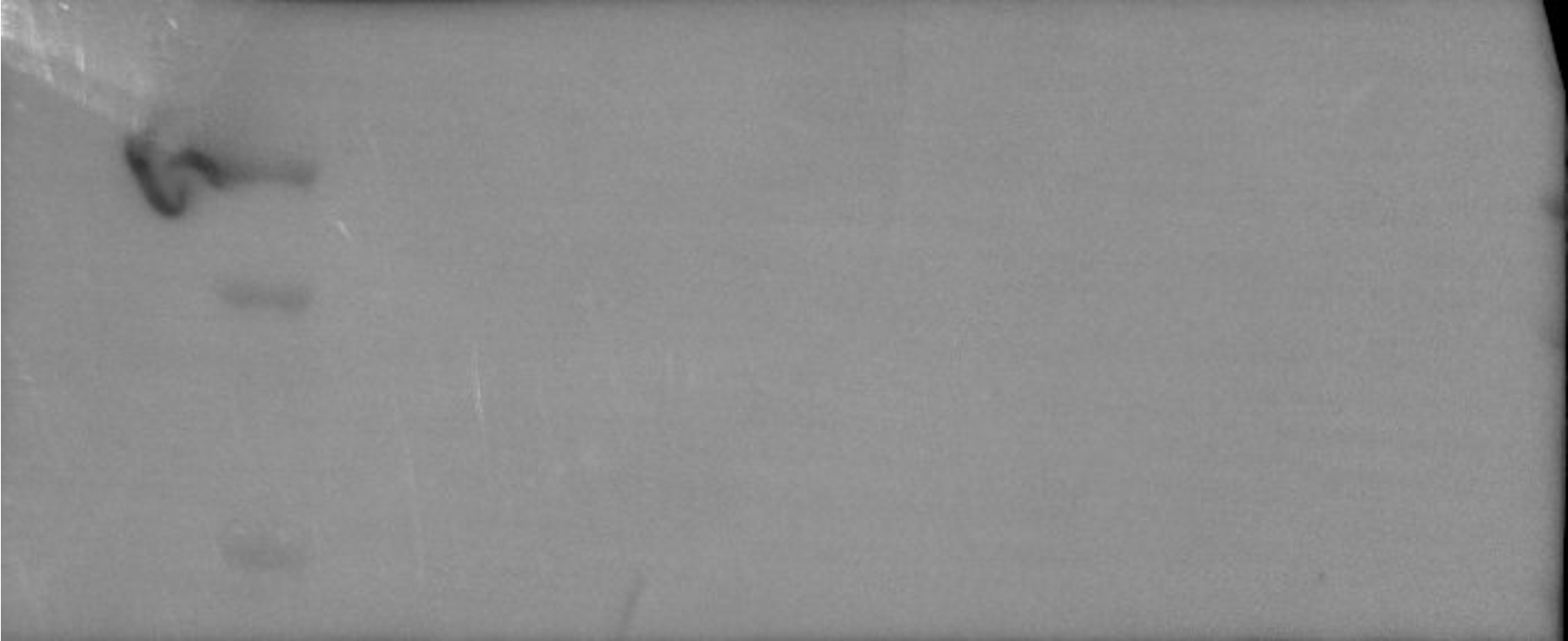

Supplement: Figure 8—source data 1. [file elife-72681-fig8-data1.zip › Figure 8 - source data/Figure 8C - source data 1 GFPxirp2 membrane.jpg]

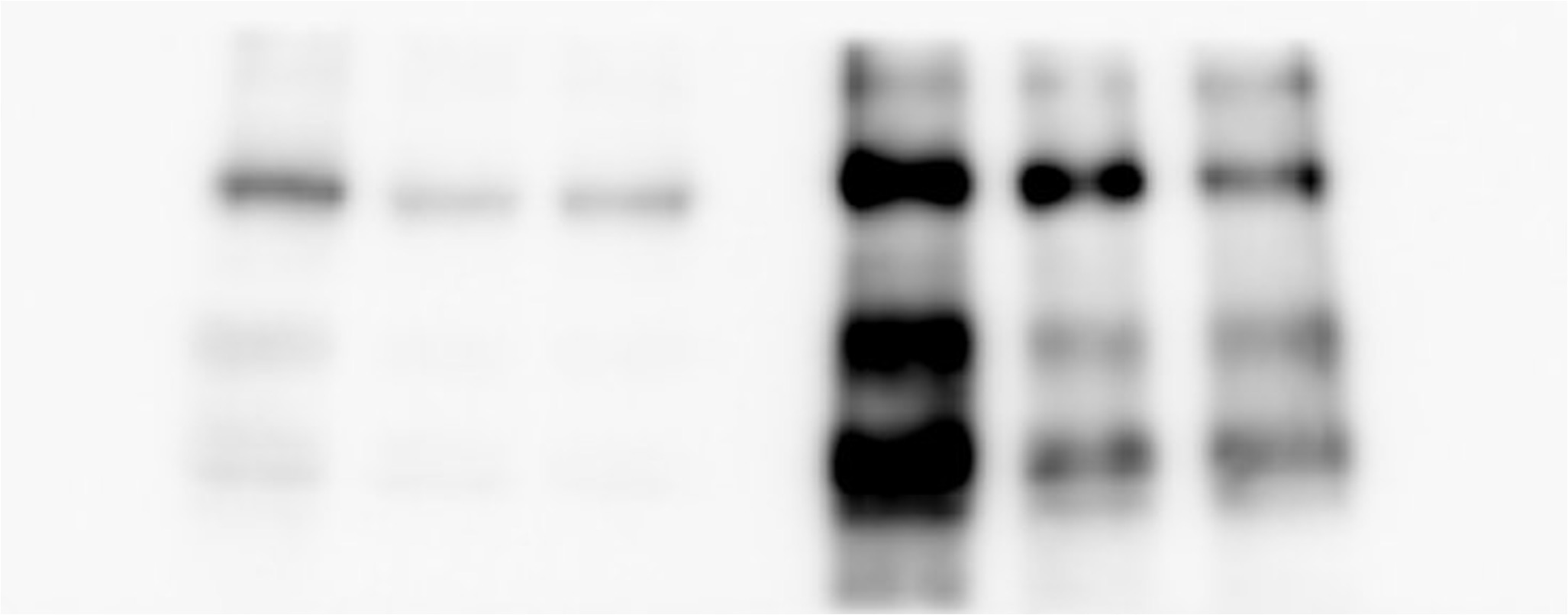

Supplement: Figure 8—source data 1. [file elife-72681-fig8-data1.zip › Figure 8 - source data/Figure 8B - source data 4 GFPxirp2 wb.jpg]

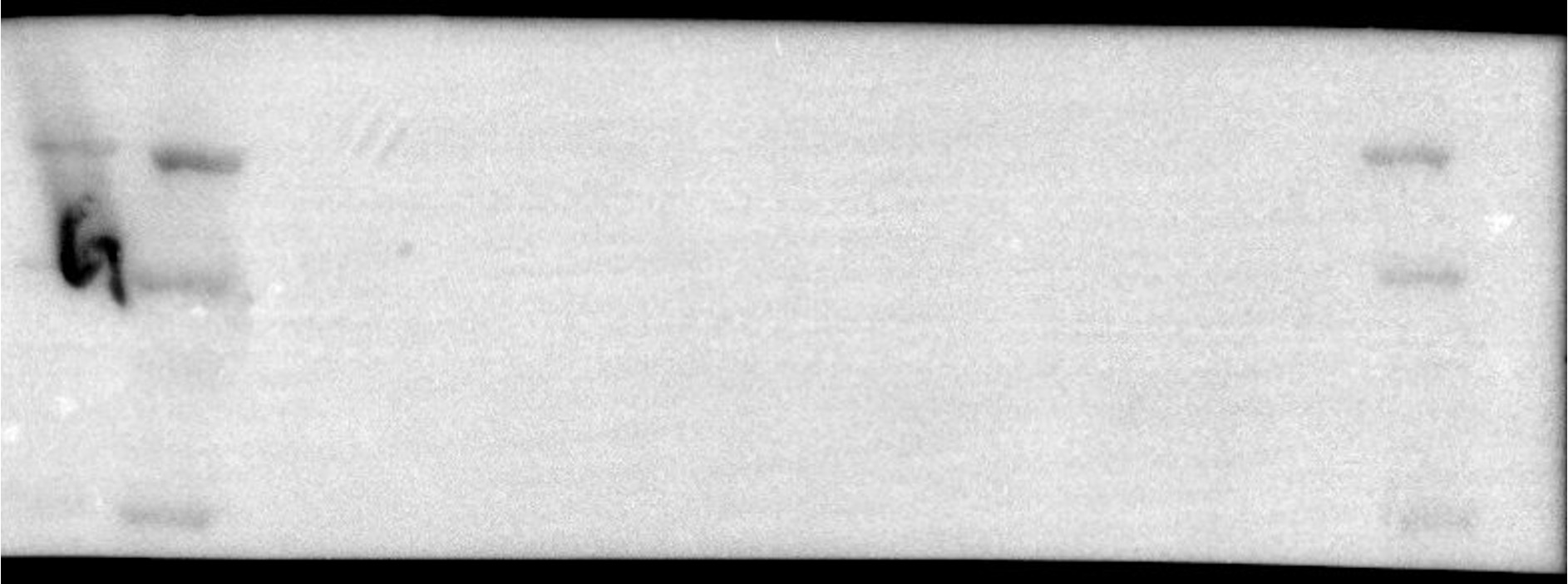

Supplement: Figure 8—source data 1. [file elife-72681-fig8-data1.zip › Figure 8 - source data/Figure 8B - source data 3 GFPxirp2 membrane.jpg]

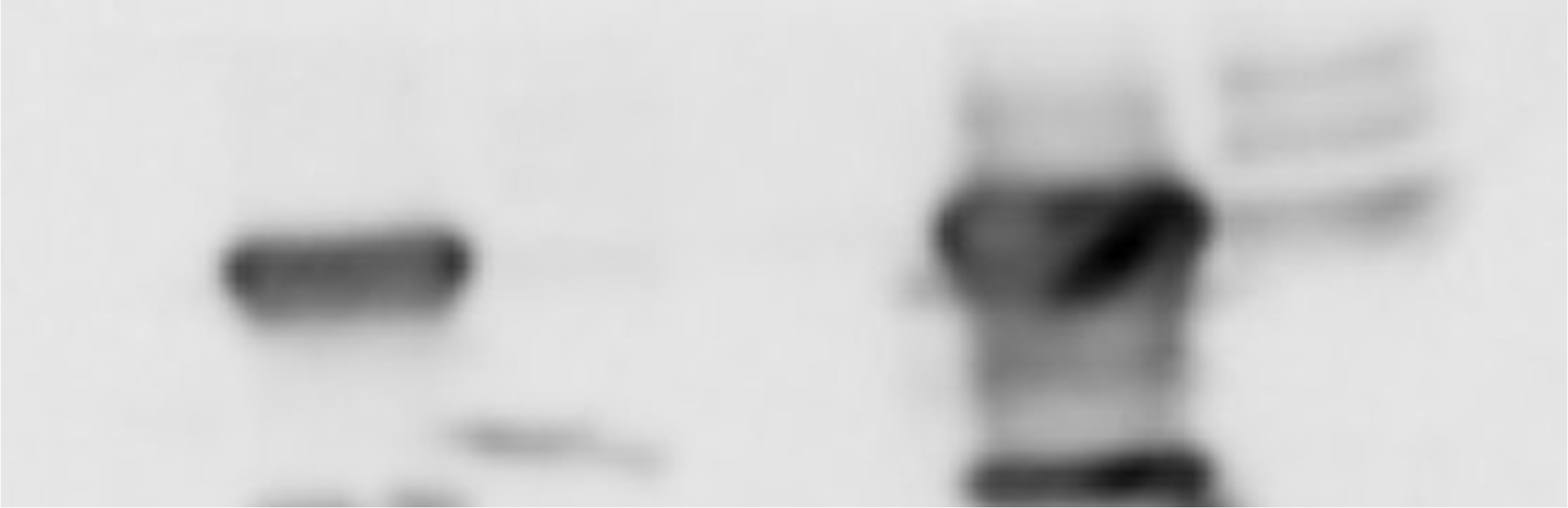

Supplement: Figure 8—source data 1. [file elife-72681-fig8-data1.zip › Figure 8 - source data/Figure 8A - source data 10 GFPshort wb.jpg]

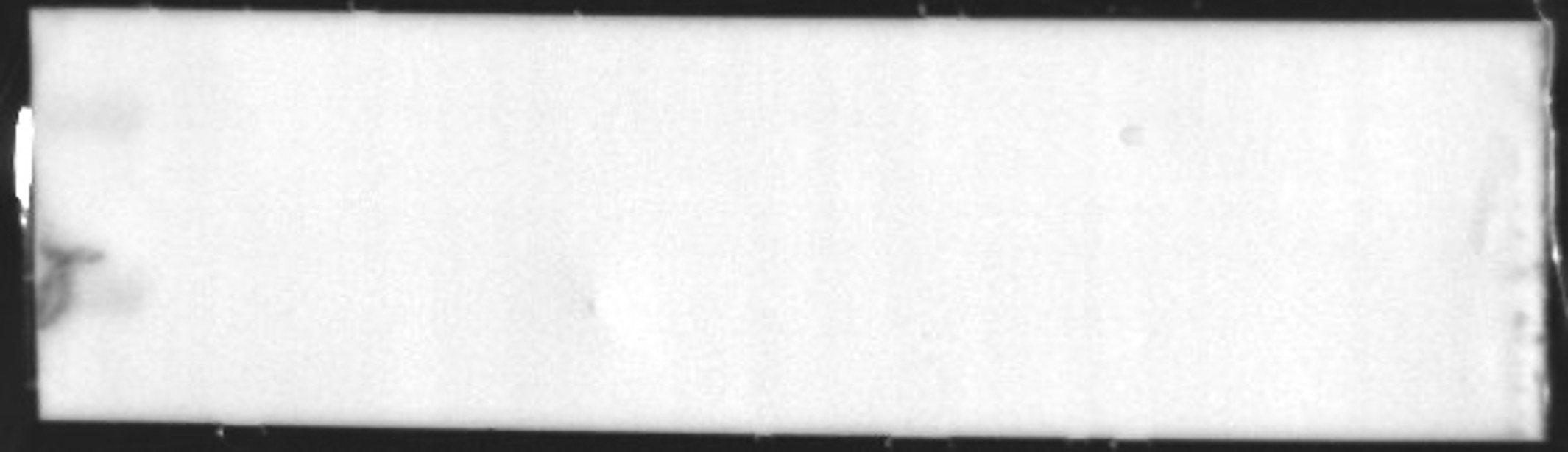

Supplement: Figure 8—source data 1. [file elife-72681-fig8-data1.zip › Figure 8 - source data/Figure 8A - source data 5 gACT membrane.jpg]

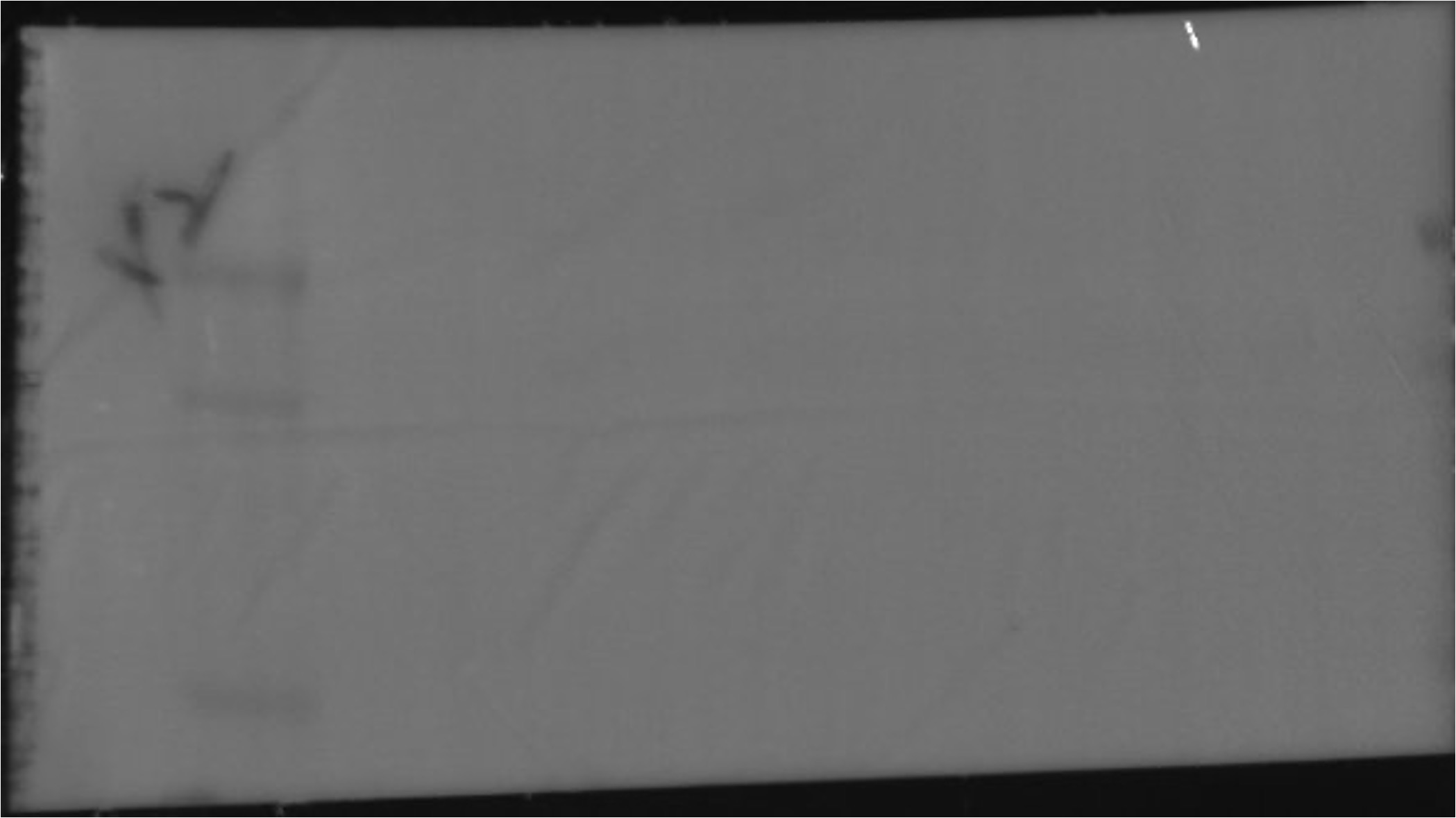

Supplement: Figure 8—source data 1. [file elife-72681-fig8-data1.zip › Figure 8 - source data/Figure 8A - source data 1 XIRP2 membrane.jpg]

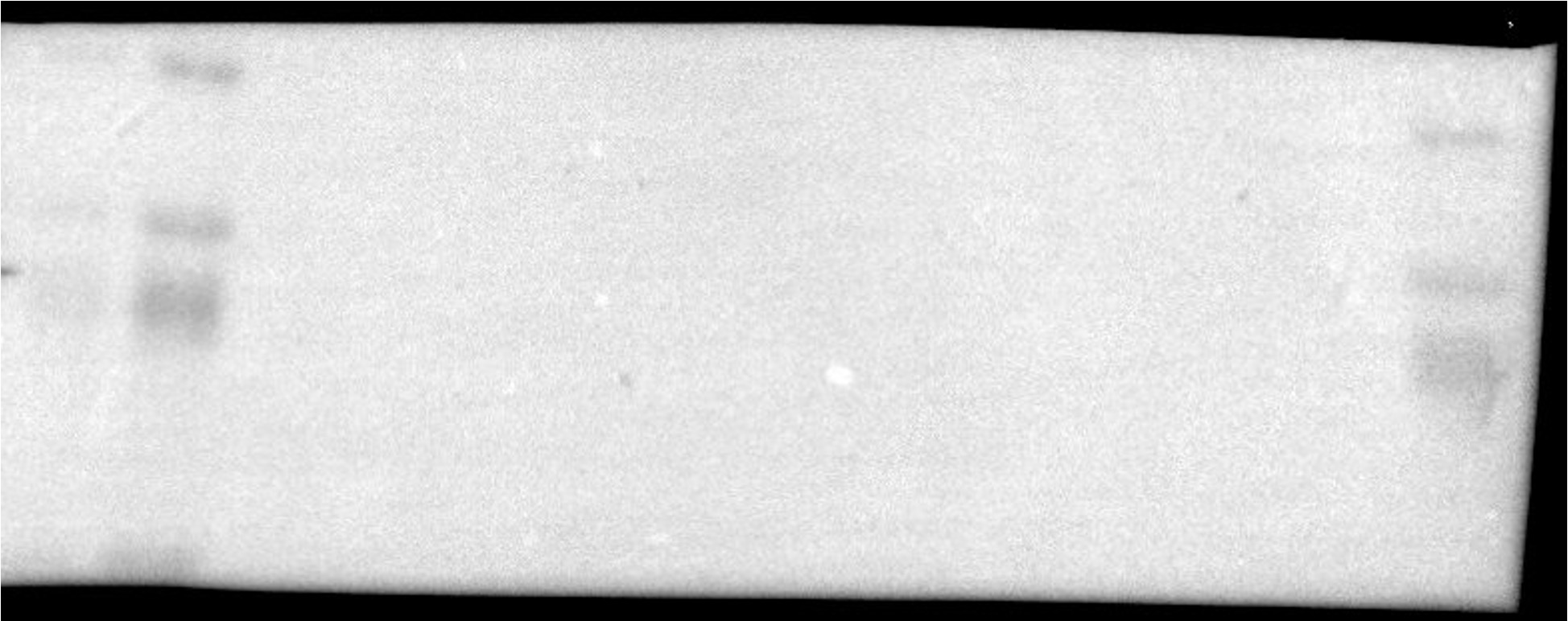

Supplement: Figure 8—source data 1. [file elife-72681-fig8-data1.zip › Figure 8 - source data/Figure 8B - source data 1 HAgACT membrane.jpg]

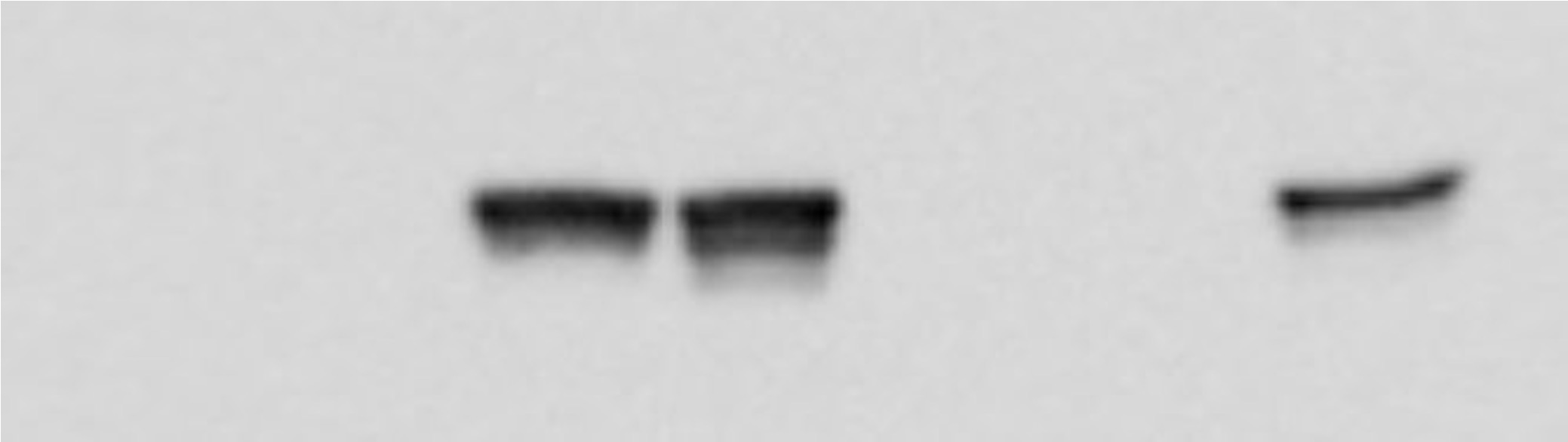

Supplement: Figure 8—source data 1. [file elife-72681-fig8-data1.zip › Figure 8 - source data/Figure 8A - source data 6 gACT wb.jpg]

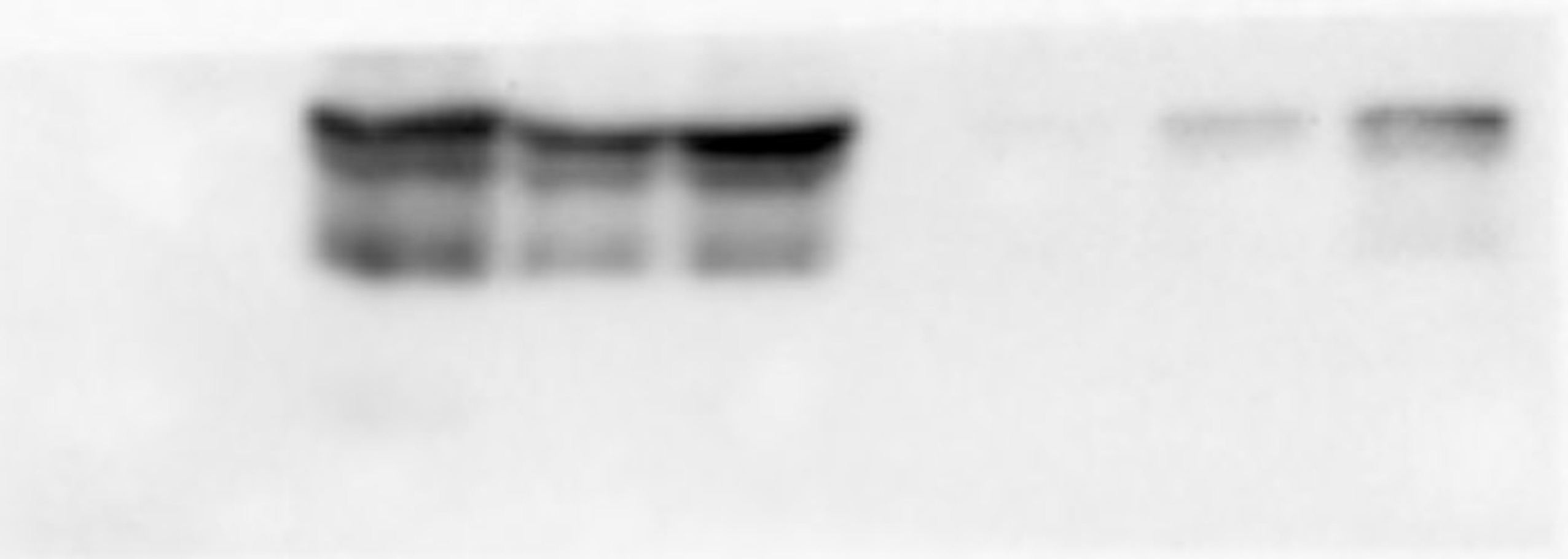

Supplement: Figure 8—source data 1. [file elife-72681-fig8-data1.zip › Figure 8 - source data/Figure 8C - source data 4 HAgACT wb.jpg]

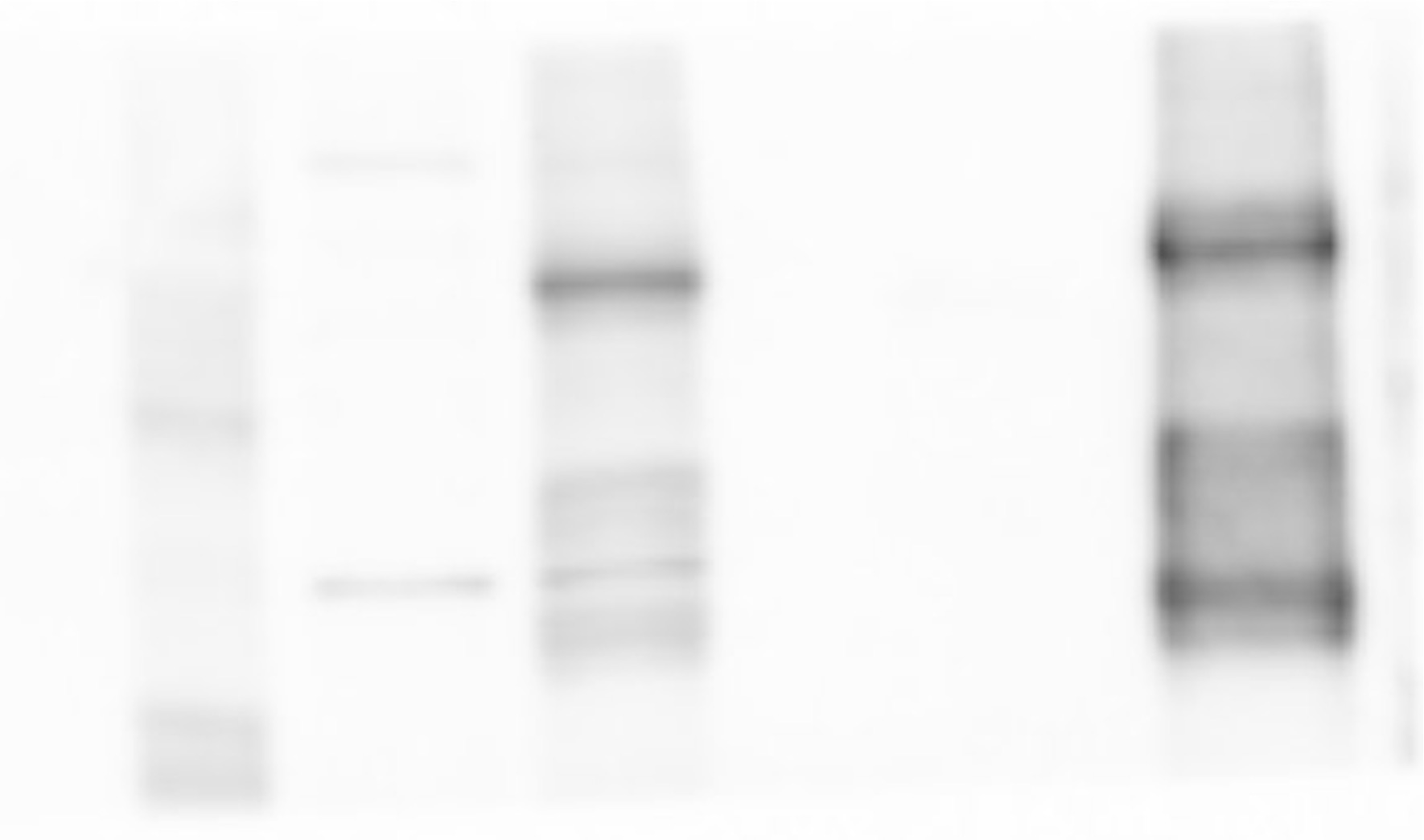

Supplement: Figure 8—source data 1. [file elife-72681-fig8-data1.zip › Figure 8 - source data/Figure 8A - source data 2 XIRP2 wb.jpg]

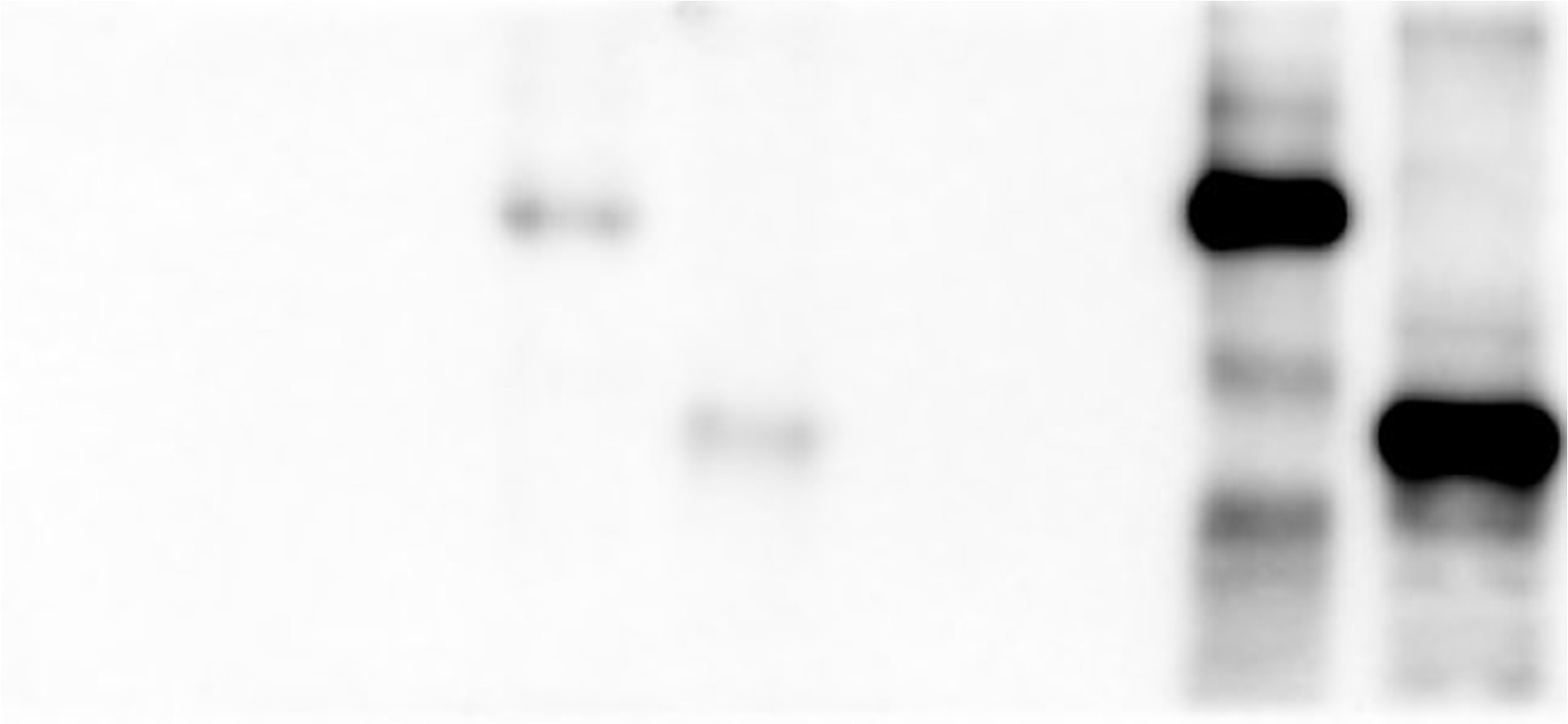

Supplement: Figure 8—source data 1. [file elife-72681-fig8-data1.zip › Figure 8 - source data/Figure 8C - source data 2 GFPxirp2 wb.jpg]

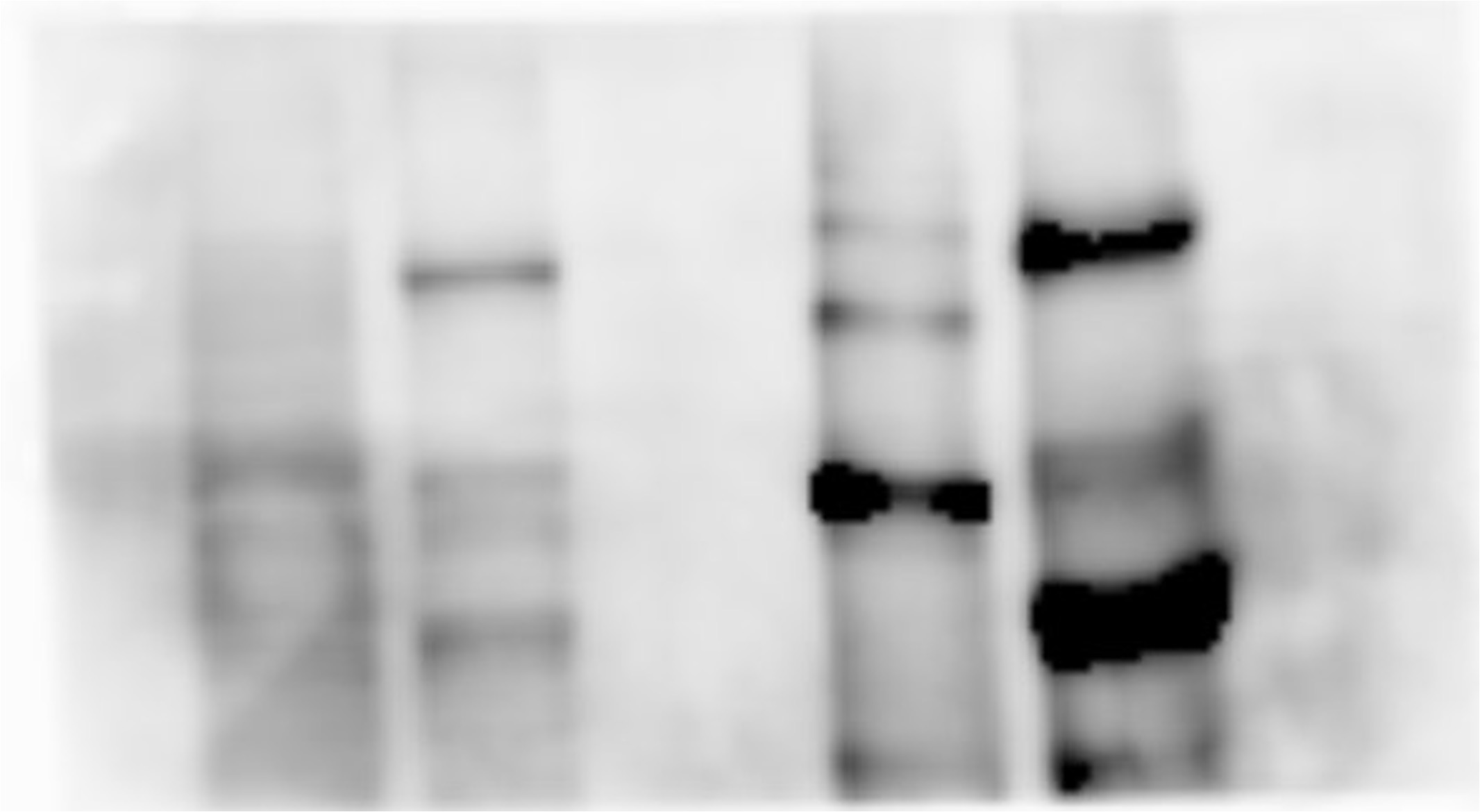

Supplement: Figure 8—source data 1. [file elife-72681-fig8-data1.zip › Figure 8 - source data/Figure 8A - source data 8 GFPlong wb.jpg]

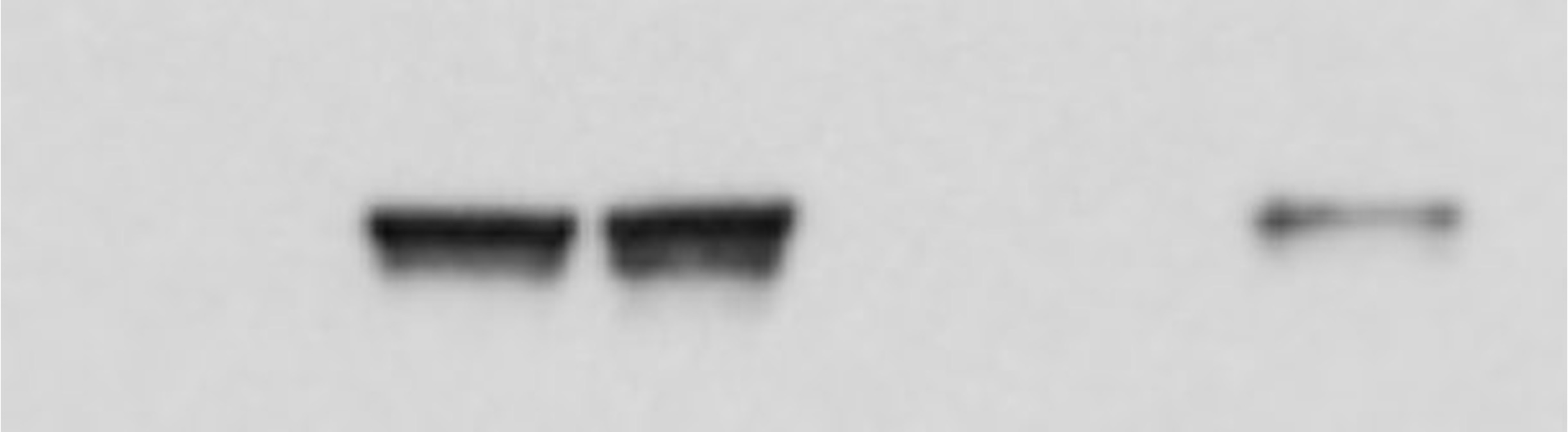

Supplement: Figure 8—source data 1. [file elife-72681-fig8-data1.zip › Figure 8 - source data/Figure 8A - source data 4 bACT wb.jpg]

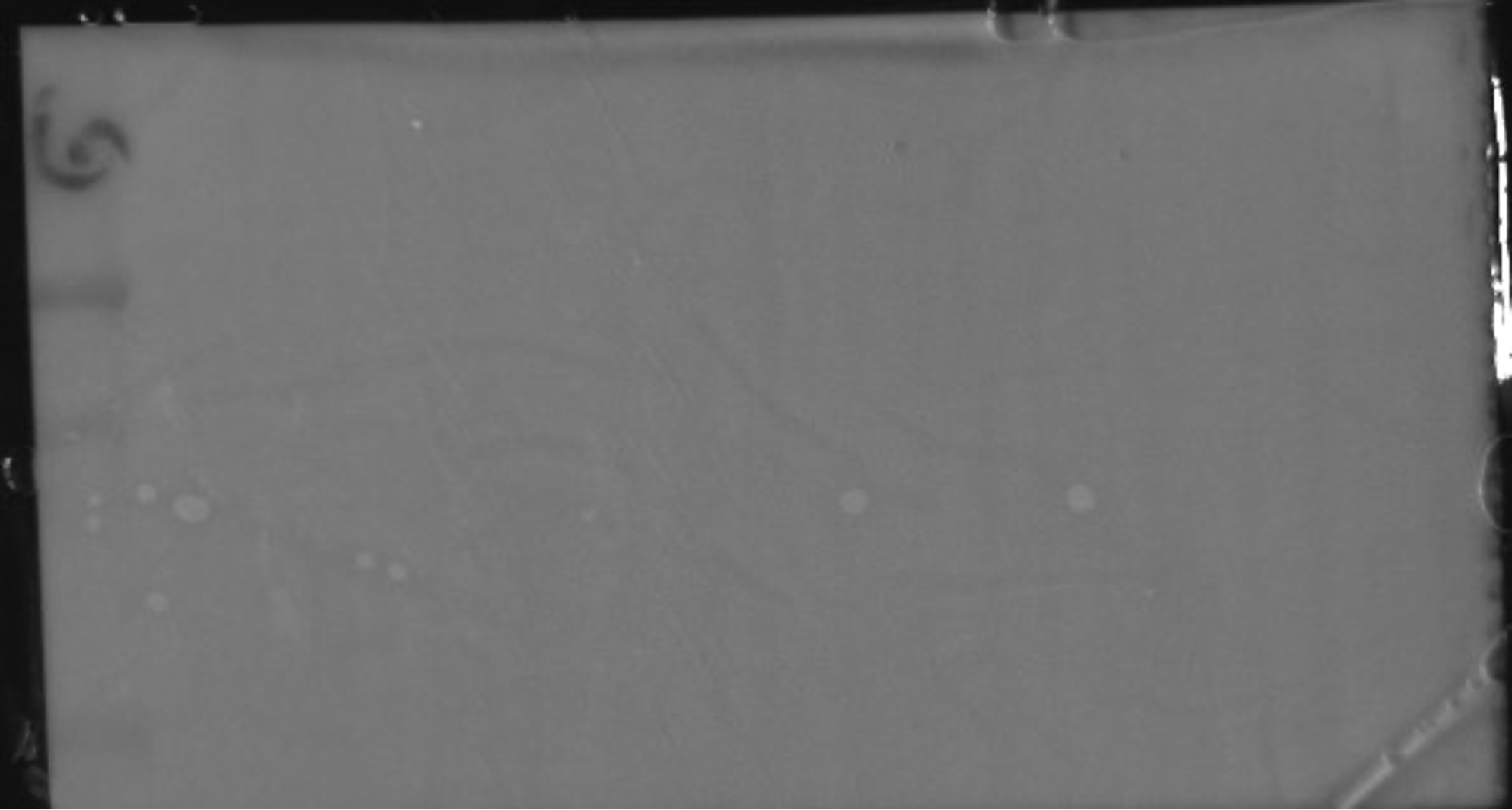

Supplement: Figure 8—source data 1. [file elife-72681-fig8-data1.zip › Figure 8 - source data/Figure 8A - source data 7 GFPlong membrane.jpg]

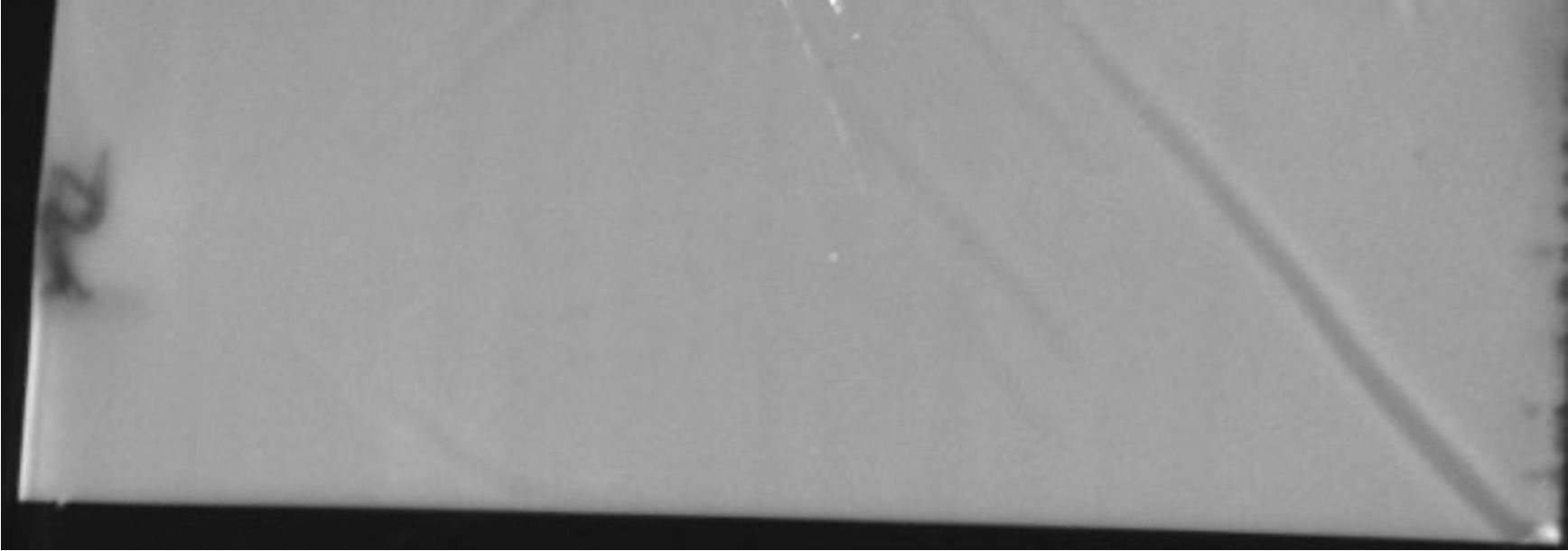

Supplement: Figure 8—source data 1. [file elife-72681-fig8-data1.zip › Figure 8 - source data/Figure 8A - source data 9 GFPshort membrane.jpg]
